# Editorial: Unraveling the mechanisms of psychiatry disorders

**DOI:** 10.3389/fnins.2026.1976981

**Published:** 2026-09-22

**Authors:** Maria Luisa Soto-Montenegro, Ana Carolina Pinheiro Campos, Diego Romero-Miguel

**Affiliations:** 1 Instituto de Investigación Sanitaria Gregorio Marañón, Madrid, Spain; 2 Centro de Investigación Biomédica en Red de Salud Mental (CIBERSAM), Instituto de Salud Carlos III, Madrid, Spain; 3 Grupo de Investigación de Alto Rendimiento en Fisiopatología y Farmacología del Sistema Digestivo de la Universidad Rey Juan Carlos (NeuGut-URJC), Madrid, Spain; 4 Hospital Sirio-Libanes, São Paulo, Brazil; 5 Centro de Biologia Molecular Severo Ochoa, Madrid, Spain

**Keywords:** addiction, depression, inflammation, molecular mechanism, oxidative stress, psychiatry, schizophrenia

Psychiatric disorders continue to resist simple biological explanations. Despite remarkable advances in neuroscience, conditions such as schizophrenia, depression, anxiety, and related neuropsychiatric syndromes are clinically recognizable yet mechanistically diffuse, emerging from interactions that span molecules, neural circuits, bodily systems, and lived experience. The contributions in this Research Topic reflect the shift from symptom-based models toward integrative frameworks that capture this complexity. Rather than converging on a single pathogenic mechanism, they frame psychiatric disorders as disturbances of interconnected biological systems whose dysfunction propagates across scales. Seven out of 11 contributions focused on schizophrenia, addressing complementary perspectives including genetics, oxidative stress, electrophysiology, sleep regulation, neural connectivity, and functional outcomes.

At the molecular level, Lin et al. identified several plasma proteins potentially involved in schizophrenia pathogenesis using proteome-wide Mendelian randomization and colocalization analyses. Proteins associated with inflammatory signaling, cellular transport, and extracellular matrix regulation, including CTSS, FOXO3, IRF3, and MMP16, were described as candidate biomarkers and possible therapeutic targets. Their results reinforce the idea that schizophrenia cannot be understood solely as a neurotransmitter disorder, but instead involves broad disruptions in immune and cellular regulatory pathways. This perspective is partially replicated by Malik et al., who examined metabolic syndrome parameters, oxidative stress markers, mitochondrial function, and glutathione-related genetic polymorphisms in drug-naïve or drug-free individuals with schizophrenia. Their study identified alterations in redox homeostasis and early metabolic vulnerability independent of antipsychotic exposure, supporting the idea that cardiometabolic dysfunction may constitute an intrinsic component of schizophrenia biology rather than merely a consequence of treatment or chronic illness. Together, these findings contribute to an increasingly consistent framework where inflammation, oxidative imbalance, and metabolic dysregulation are deeply associated within psychiatric disease processes. Extending this systemic perspective, Yan et al. explored the relationship between ventricular cardiac structure and schizophrenia risk from a “heart-to-brain” genetic perspective. Their Mendelian randomization analyses provided evidence consistent with a potential association between genetically predicted higher right ventricular end-systolic volume and lower schizophrenia risk. Although their results are still preliminary, they point an emerging trend in psychiatry, where genetically informed analysis may reveal links between peripheral physiological traits and neuropsychiatric vulnerability.

At a circuit level, Wang et al. investigated functional and causal connectivity in first-episode, drug-naïve early-onset schizophrenia. Their findings point toward disrupted cortico-striatal-cerebellar communication, including aberrant connectivity involving the caudate nucleus, prefrontal cortex, and default mode network regions. This association between altered caudate-precuneus connectivity and delusional symptom severity was particularly relevant, reinforcing models that conceptualize psychosis as a disorder of large-scale network integration rather than isolated regional dysfunction. A related topic appears in the electrophysiological study by Sun et al., who examined face processing using high-density EEG. Interestingly, patients displayed enhanced N170 amplitudes in response to faces despite reduced alpha-band phase locking. Rather than reflecting preserved processing, this pattern suggests a hyper-responsive yet inefficient compensatory mechanism in socially relevant perceptual systems. Such findings complicate simplistic deficit models of schizophrenia by showing that increased neural activation may coexist with impaired temporal coordination and reduced processing precision. This disruption in temporal organization also appears in the study by You et al., who explored the relationship between cognitive insight, stigma, dysfunctional sleep beliefs, and chronotype in schizophrenia. Their mediation analyses suggested that heightened cognitive insight may paradoxically contribute to eveningness chronotype tendencies through increased stigma internalization and maladaptive sleep-related cognitions. Beyond the specific findings, their study describes how biological rhythms, cognition, and psychosocial experience interact dynamically in psychiatric disorders, challenging artificial separations between “psychological” and “biological” domains. The multidimensional consequences of schizophrenia are further highlighted in the review by Ifteni et al., which examines nearly five decades of functionality assessment scales. Their analysis underscores a longstanding difficulty within psychiatry: symptom reduction alone does not adequately capture recovery. Functional outcomes, including autonomy, interpersonal relationships, occupational engagement, and social integration, remain central yet incompletely measured dimensions of psychiatric care.

Although schizophrenia occupies a substantial portion of this Research Topic, the included studies collectively extend beyond diagnostic boundaries. Depression, anxiety, neurodegeneration, chronic disease, and social adversity repeatedly intersect through shared biological pathways. Untu et al. examine the complex relationship between depression and Alzheimer's disease dementia, highlighting common mechanisms involving neuroinflammation, neurotransmitter dysregulation, hypothalamic-pituitary-adrenal axis alterations, and impaired neuroplasticity. Their review highlights how psychiatric symptoms in neurodegenerative disorders cannot be reduced to secondary psychological reactions, but may instead emerge from partially overlapping pathogenic substrates. Similarly, Qaisiya et al. explore social isolation and loneliness as biologically embedded phenomena capable of disrupting cellular homeostasis through oxidative stress, inflammation, mitochondrial dysfunction, endocrine dysregulation, and impaired autophagy. Their synthesis is particularly timely in the post-pandemic era, where social disconnection increasingly appears not merely as a psychosocial condition but as a biologically consequential stress state with psychiatric and metabolic implications. The translational implications of mechanistic psychiatry are especially evident in the contributions focused on therapeutic innovation. Pu et al. compared intermittent theta-burst stimulation and sequential bilateral repetitive transcranial magnetic stimulation in major depressive disorder. While both interventions produced clinical benefit, intermittent theta-burst stimulation induced greater improvements in depressive symptoms and cognitive reappraisal, supporting the possibility that distinct neuromodulatory protocols engage partially different prefrontal plasticity mechanisms. These findings exemplify the gradual shift toward circuit-informed therapeutic approaches in psychiatry. Complementing this perspective, Yuan et al. reviewed mechanistic and clinical evidence regarding curcumin as an adjunctive intervention for depression and anxiety associated with chronic diseases. Across both clinical and preclinical studies, curcumin appeared to influence inflammatory, oxidative, neuroplastic, and gut-related pathways implicated in affective symptomatology. Although methodological heterogeneity remains substantial, this review reflects growing interest in therapies capable of simultaneously targeting multiple convergent biological mechanisms.

Collectively, the studies in this Research Topic illustrate the complexity of psychiatric disorders and the diverse approaches used to investigate their biological basis. Despite addressing different diagnoses and methodologies, several common themes emerge, including the involvement of immune and metabolic pathways, alterations in brain connectivity and electrophysiological function, and the search for biomarkers and novel therapeutic strategies. Together, they reinforce the idea that psychiatric disorders arise from interacting biological processes rather than alterations within a single system. However, major challenges remain, particularly in understanding the relationship between peripheral and central dysfunctions, and integrating molecular findings with neuroimaging, electrophysiology and clinical findings. Most importantly, the field still lacks longitudinal evidence that would help clarify how these different mechanisms contribute to disease onset, progression, and treatment response. We hope this Research Topic provides a useful overview of these emerging directions and encourages further studies that integrate different levels of biological and clinical investigation. Progress in understanding psychiatric disorders will probably not come from identifying a single underlying mechanism, but from clarifying how different pathways interact across individuals and across stages of disease.

