# Correction: Editorial: Unraveling the mechanisms of psychiatry disorders

**DOI:** 10.3389/fnins.2026.2007900

**Published:** 2026-10-12

**Authors:** Maria Luisa Soto-Montenegro, Ana Carolina Pinheiro Campos, Diego Romero-Miguel

**Affiliations:** 1 Instituto de Investigación Sanitaria Gregorio Marañón, Madrid, Spain; 2 Centro de Investigación Biomédica en Red de Salud Mental (CIBERSAM), Instituto de Salud Carlos III, Madrid, Spain; 3 Grupo de Investigación de Alto Rendimiento en Fisiopatología y Farmacología del Sistema Digestivo de la Universidad Rey Juan Carlos (NeuGut-URJC), Madrid, Spain; 4 Hospital Sirio-Libanes, São Paulo, Brazil; 5 Centro de Biologia Molecular Severo Ochoa, Madrid, Spain

**Keywords:** addiction, depression, inflammation, molecular mechanism, oxidative stress, psychiatry, schizophrenia

An incorrect citation for Ana Carolina Pinheiro Campos was provided. The correct citation reads: Campos ACP

An incorrect abbreviation in the author contribution section for Maria Luisa Soto-Montenegro and Ana Carolina Pinheiro Campos was provided. The correct abbreviations reads: MLS-M and ACPC.

An incorrect Funding statement was provided. The correct funding statement reads:

The author(s) declared that financial support was received for this work and/or its publication. This work was funded by grant PID2021-128862OB-I00 funded by MICIU/AEI/10.13039/501100011033 and by FEDER, UE; Ministerio de Ciencia, Innovación y Universidades, Instituto de Salud Carlos III (ISCIII), through the project “CIBER-Consorcio Centro de Investigación Biomédica en Red CB07/09/0031”; Delegación del Gobierno para el Plan Nacional sobre Drogas correspondiente a fondos del Mecanismo de Recuperación, Transformación y Resiliencia de la Unión Europea (project EXP2022/008917), Delegación del Gobierno para el Plan Nacional sobre Drogas (project 2024I090) and II Edition of iSanidad y Herbalife Nutrition Grant.

The original version of this article has been updated.

